# IGF-1 protects retinal ganglion cells from hypoxia-induced apoptosis by activating the Erk-1/2 and Akt pathways

**Published:** 2013-09-12

**Authors:** Xuesen Yang, Aimin Wei, Yong Liu, Genlin He, Zhou Zhou, Zhengping Yu

**Affiliations:** 1 Department of Occupational Health, Third Military Medical University, Chongqing, China; 2 Institute of Tropical Medicine, Third Military Medical University, Chongqing, China; 3 Southwest Hospital, Southwest Eye Hospital, Third Military Medical University, Chongqing, China

## Abstract

**Purpose:**

Hypoxia-induced retinal ganglion cell (RGC) apoptosis has been implicated in many optic neuropathies. Insulin-like growth factor-1 (IGF-1) is important in maintaining neuronal survival, proliferation, and differentiation. The purpose of this study is to explore whether IGF-1 can protect RGCs from hypoxia-induced apoptosis and to determine the precise mechanisms that regulate this process.

**Methods:**

Purified RGC cultures were obtained from the retinas of neonatal Sprague Dawley (SD) rats using a two-step panning method. Primary cultured RGCs were cultured in a closed hypoxic chamber (5% O_2_, 5% CO_2_, and 90% N_2_) for 12 h with or without IGF-1. The degree of apoptosis in the RGCs was detected by caspase-3 expression and TUNEL and JC-1 staining assays. The expression and phosphorylation of protein kinase B (Akt), p44/42 mitogen-activated protein kinase (MAPK) (extracellular signal-regulated kinase-1/2 [Erk-1/2]), Bad, and caspase-3 was investigated with immunoblot analysis.

**Results:**

Hypoxia induces apoptosis in primary Sprague Dawley rat RGCs, as detected by caspase-3 expression and TUNEL and JC-1 staining assays, and that IGF-1 treatment could significantly reduce this effect in RGCs. Interestingly, pretreatment of RGCs with AG1024 (an IGF-1 inhibitor), U0126 (an Erk-1/2 inhibitor), and LY294002 (an Akt inhibitor) markedly attenuated the effects of IGF-1 treatment. Furthermore, western blot analysis suggested that the Erk-1/2 and Akt signaling pathways play a role in the protective effects of IGF-1 on RGCs exposed to hypoxia.

**Conclusions:**

These data indicate that IGF-1 can protect primary cultured RGCs against hypoxia-induced apoptosis via the Erk-1/2 and Akt signaling pathways, suggesting that IGF-1 treatment is a potential therapeutic approach for treating hypoxia-induced neurodegeneration in the retina.

## Introduction

Hypoxia is a critical pathological factor in several retinal diseases including central retinal artery occlusion, glaucoma, diabetic retinopathy, hypertensive vascular disease, and ischemic central retinal vein thrombosis [1-3]. Retinal hypoxia associated with these diseases is a common cause of visual impairment and blindness [4]. Retinal ganglion cells (RGCs), the sole neurons that can relay visual signals to the brain and therefore play a crucial role in the visual system, are particularly sensitive to hypoxic stress [5]. Hypoxia can induce RGC apoptosis [6,7], which is the major cause of progressive vision loss in sight-threatening disorders [8,9]. Although the exact mechanisms underlying hypoxia-induced apoptosis in RGCs remain unclear, it is hypothesized that protecting RGCs against hypoxia-induced apoptosis may be beneficial for treating hypoxia-induced diseases of the retina.

Insulin-like growth factor 1 (IGF-1), a single-chain peptide of 70 amino acids with considerable homology to insulin, plays an important role in normal brain development, neuronal growth, and cellular proliferation and differentiation. It has also been demonstrated that IGF-1 can have neuroprotective effects in neurons that are subject to several stressors, including hypoxia-ischemia [10,11]. Several experimental studies have shown that IGF-1 protects RGCs from death and promotes the regeneration of axons following damage to the optic nerve (ON) in adult rats and goldfish [12,13]. Moreover, IGF-1 inhibits the death of neuroretinal cells in diabetic rats [14]. These data suggest that IGF-1 may exert neuroprotective effects for RGCs survival. However, how IGF-1 protects against hypoxia-induced apoptosis in RGCs is unclear.

IGF-1 binds to the IGF-1 receptor (IGF-1R) and exerts its biologic actions by activating several intracellular signaling cascades, such as the extracellular signal-regulated kinase 1/2 (Erk-1/2) and phosphatidylinositol 3-kinase (PI3K)/ protein kinase B (Akt) pathways, in various cell types [15]. The survival-promoting effects of IGF-1 are executed, at least in part, through the activation of the PI3K/Akt pathway [16]. Nevertheless, the Akt response to hypoxia seems to be cell type-specific. For example, hypoxia can induce the activation of the PI3K/Akt cell survival pathway in PC12 cells and HeLa cells [17,18], but not in mouse 3T3 cells [19]. Meanwhile, the role of Erk-1/2 in apoptosis is controversial. Increasing evidence suggests an emerging role of Erk-1/2 in supporting neuronal survival [20-22]; however, a study by Zhuang et al. identified activated Erk-1/2 as a proapoptotic factor under certain conditions such as ischemia-induced brain injury [23]. Moreover, previous studies have indicated that the PI3K/Akt and Erk-1/2 intracellular pathways mediate the response of IGF-1 in axotomized RGCs [24]. However, the intracellular signaling pathways involved in IGF-1-mediated protection against hypoxia-induced apoptosis in RGCs have not been characterized. Therefore, we used a multigas incubator to induce hypoxia in the primary cultured RGCs and observed the changes in cellular activity and Erk1/2 and Akt levels to explore the precise intracellular signaling pathways.

## Methods

### Materials

Cell culture reagents were obtained from HyClone (Logan, UT). Thy-1.1 was purchased from Chemicon (MAB1406; Temecula, CA). Goat antimouse second antibody was obtained from ZSBIO (ZDR-5307; Beijing, China). Poly-L-lysine solution was purchased from Sigma (P8920; St. Louis, MO). The in situ cell death detection kit-POD was purchased from Roche (No. 11684817910; Indianapolis, IN), and Hoechst 33342 was purchased from Molecular Probes (Eugene, OR). The fluorescent, lipophilic, and cationic probe (5,5’,6,6’-tetrachloro1, 1’,3,3’-tetraethyl-benzimidazolyl carbocyanine iodide; JC-1; C2006) and the caspase-3 activity assay kit were purchased from Beyotime (C1115; Shanghai, China). Recombinant rat IGF-1 was obtained from Abcam (ab52006; Cambridge, UK). The phosphatase inhibitor cocktail B (sc-45045), C (sc-45065), and phospho-Bad (Ser136) antibody (sc-12969) were purchased from Santa Cruz Biotechnology (Santa Cruz, CA). The rabbit polyclonal Akt antibody (9272), mouse monoclonal phospho-Akt antibody (4051), mouse monoclonal p44/42 mitogen-activated protein kinase (MAPK) (Erk-1/2) antibody (4696), rabbit monoclonal phospho-p44/42 MAPK (Erk-1/2) antibody (4370), and rabbit polyclonal phospho-Bad (Ser112) antibody (9291) were obtained from Cell Signaling Technology (Beverly, MA). The mouse monoclonal β-actin antibody (A1978) and Akt inhibitor LY294002 (L9908) were obtained from Sigma. The IGF-1 reporter inhibitor AG1024 (121,767) and the Erk-1/2 inhibitor U0126 (662,005) were purchased from Calbiochem (La Jolla, CA). Special Odyssey blocking buffer and secondary antibodies, including IRDye 680 donkey antirabbit antibody (926–32223) and IRDye 800 donkey anti-mouse antibody (926–32212), were obtained from LI-COR (Lincoln, NE).

### Cell culture

RGCs were prepared according to the method described previously, with minor modifications [25,26]. All of the procedures described in this study were performed in accordance with the Guide for the Care and Use of Laboratory Animals – Chinese Version (1996). Briefly, neonatal Sprague Dawley rats were killed by decapitation, and their eyes were rapidly removed and immersed in a calcium- and magnesium-free (CMF) salt solution (0.1 M Dulbecco's phosphate-buffered saline [PBS]; calcium- and magnesium-free; Gibco, Grand Island, NY). Approximately 30 eyes were harvested for each experiment. The retinas were rapidly isolated and incubated at 37 °C for 25 min in CMF containing 0.1% trypsin. Then, cells were incubated for 5 min with a mouse antimacrophage antibody. The cell suspensions were then incubated for 30 min on a Petri dish coated with a goat antimouse immunoglobulin G (H + L chain) antibody. Suspensions containing cells that did not adhere to the Petri dish were harvested and incubated for 1 h in a dish coated with an anti-Thy-1.1 antibody. The cells that adhered to the dish were then trypsinized (0.1% trypsin for 10 min), after which they were diluted to 1 x 10^5^ cells/ml and placed on dishes or glass coverslips that had previously been coated with 50 mg/ml poly-L-ornithine. Cells were incubated in a serum-free culture medium, which was prepared using B27-supplemented neurobasal medium. The cultures were maintained in a humidified atmosphere of 5% CO_2_ and 95% air at 37 °C, and the medium was changed every 3 days. Cells were used after 5 days.

### Hypoxic injury to retinal ganglion cells

Hypoxia was induced according to the study by Hong et al. [20]. Briefly, cultures were transferred into an automatically controlled Multi Gas Incubator (YCPHOSPHO- 50S, BaiDianTech, Hua-xi, Changsha, China), in which the oxygen levels (5% O_2_, 5% CO_2_, and 90% N_2_) and temperature (37 °C) were maintained. After the cells were washed twice with deoxygenated serum-free DMEM, they remained in the hypoxic incubator for various incubation durations. A vacuum de-aerator is used to remove oxygen from the modified Dulbecco's Modified Eagle's Medium (DMEM; Gibco), thus the deoxygenated serum-free DMEM was obtained. After washing twice with deoxygenated serum-free DMEM, cells were maintained in the hypoxic incubator for various incubation durations. Based on the literature and preexperimental findings [27-30], the various IGF-1 concentrations were added to the same medium 6 h before hypoxic injury. AG1024 (10 uM), U0126 (5 uM), and LY294002 (20 uM) were added to the culture media 1 h before IGF-1 was administered. Control cells were not exposed to hypoxia.

### TUNEL assay

The terminal deoxynucleotidyl transferase-mediated uridine 5′-triphosphate-biotin nick end labeling (TUNEL) assay was conducted according to the manufacturer’s protocol. Briefly, at the end of the 5-day culture period, RGCs on coverslips were fixed in 4% paraformaldehyde overnight at 4 °C and permeabilized in 0.1% Triton X-100 in 0.1% sodium citrate for 2 min. They were subsequently incubated for 1 h at 37 °C in a TUNEL reaction medium that specifically identified apoptotic nuclei by labeling fragmented DNA with the fluorochrome, fluorescein isothiocyanate. After rinsing in 0.01 M phosphate buffered saline (PBS, pH 7.4), the nuclei of the labeled RGCs were stained with 5 μg/ml Hoechst 33342 for 5 min. The cells were then mounted on microscope slides and examined under ultraviolet light using an epifluorescence microscope. Nuclei that showed green fluorescence were counted as apoptotic; the total nuclear number of blastocysts was determined based on the Hoechst 33342 staining. Approximately ten visual fields were analyzed in each replicate; the experiment was replicated three times.

### Measurement of mitochondrial membrane potential

The fluorescent, lipophilic, and cationic probe JC-1 measured the mitochondrial membrane potential (ΔΨm) of RGCs according to the manufacturer’s directions. Briefly, after the indicated treatments, the cells were cultured in 24-well plates and incubated with the JC-1 staining solution (5 μg/ml) for 20 min at 37 °C. The cells were then rinsed twice with JC-1 staining buffer, and the fluorescence intensity of the mitochondrial JC-1 monomers (λex 514 nm, λem 529 nm) and aggregates (λex 585 nm, λem 590 nm) was detected using a monochromator microplate reader (Safire II; Tecan, Maenneddorf, Switzerland). The ΔΨm of the RGCs in each treatment group was calculated as the fluorescence ratio of red (i.e., aggregates) to green (i.e., monomers). The experiment was replicated three times.

### Caspase-3 activity assay

Caspase-3 protease activity in the cells was measured using a caspase-3 colorimetric assay kit, according to the manufacturer's instructions. Briefly, the cells were collected, rinsed with ice-cold PBS, and lysed with lysis buffer on ice. The cell lysate was centrifuged (12,000 ×*g*, 10 min, 4 °C). The resulting supernatants were used as the cell extracts. Protein concentrations were determined using the Lowry protein assay with bovine serum albumin as the standard. The supernatant was incubated with the acetyl-Asp-Glu-Val-Asp p-nitroanilide (Ac-DEVD-pNA) substrate and reaction buffer for 2 h at 37 °C. The levels of the chromophore p-nitroanilide (pNA) released by caspase-3 activity were spectrophotometrically quantified. The data were normalized for protein concentration. The experiment was replicated three times.

### Western blot analysis

Protein samples (60 μg) were separated by different concentrations of sodium dodecyl sulfate–polyacrylamide gel electrophoresis, transferred to nitrocellulose membranes, and blocked with Odyssey blocking buffer at room temperature for 1 h. Immunoblots were incubated at 4 °C overnight with primary antibodies specific for Akt (1:1,000), phospho-Akt (1:1,000), p44/42 MAPK (Erk-1/2; 1:2,000), phospho-p44/42 MAPK (Erk-1/2; 1:2,000), phospho-Bad (Ser136; 1:400), phosphor-Bad (Ser112; 1:1,000), and caspase-3 (1:1,000). After washing with Tris-Buffered Saline Tween-20 (TBST), the membranes were incubated with secondary antibody (1:5,000) for 30 min at room temperature with gentle shaking. After a final wash with PBS, the signal was then detected and quantified using the Odyssey infrared imaging system (LI-COR) and normalized to the reference bands of β-actin.

### Statistical analysis

All data are expressed as the mean percentage of the control value plus the standard error of the mean (SEM) for the indicated number of experiments, and each experiment was repeated at least three times. The overall statistical significance was first obtained with one-way analysis of variance (ANOVA). The statistical significance of all pairs of multiple groups of data was assessed using the Newman–Keuls comparison test. A value of p<0.05 was considered significant.

## Results

### Immunocytochemical identification of cultured retinal ganglion cells

**Figure 1:**
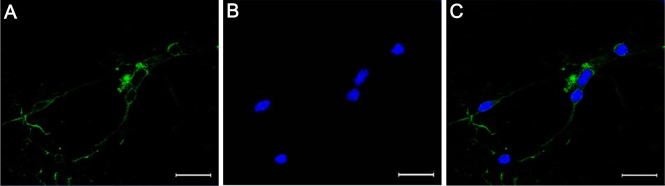
Immunocytochemical images of cultured retinal ganglion cells. Positive retinal ganglion cells (RGCs) were stained green with Thy-1.1, and their nuclei were stained blue with Hoechst 33342. **A**: Fluorescence microscope image of the retinal ganglion cell after labeling with Thy-1.1 (in green). **B**: Fluorescence microscope image of the retinal ganglion cell after labeling with Hoechst 33342 (in blue). **C**: Merged image of (**A**) and (**B**). Bar: 50 μm.

To verify that the RGCs were isolated from all other retinal cell types, the isolated retinal cells were immunostained with the primary antibody specific for RGCs, Thy-1.1, on the third day after plating (Figure 1). Cultured RGCs have round or oval cell bodies with a diameter of 20–30 µm and branched neurites of uniform caliber and varying length. In the culture wells containing panned cells, an average of 95.9% (±3%) of cells were positive for Thy-1.1 staining, showing that the cultured RGCs showed a high immunopositive rate for Thy-1.1.

### IGF-1 reduces hypoxia-induced apoptosis in primary cultured retinal ganglion cells

**Figure 2:**
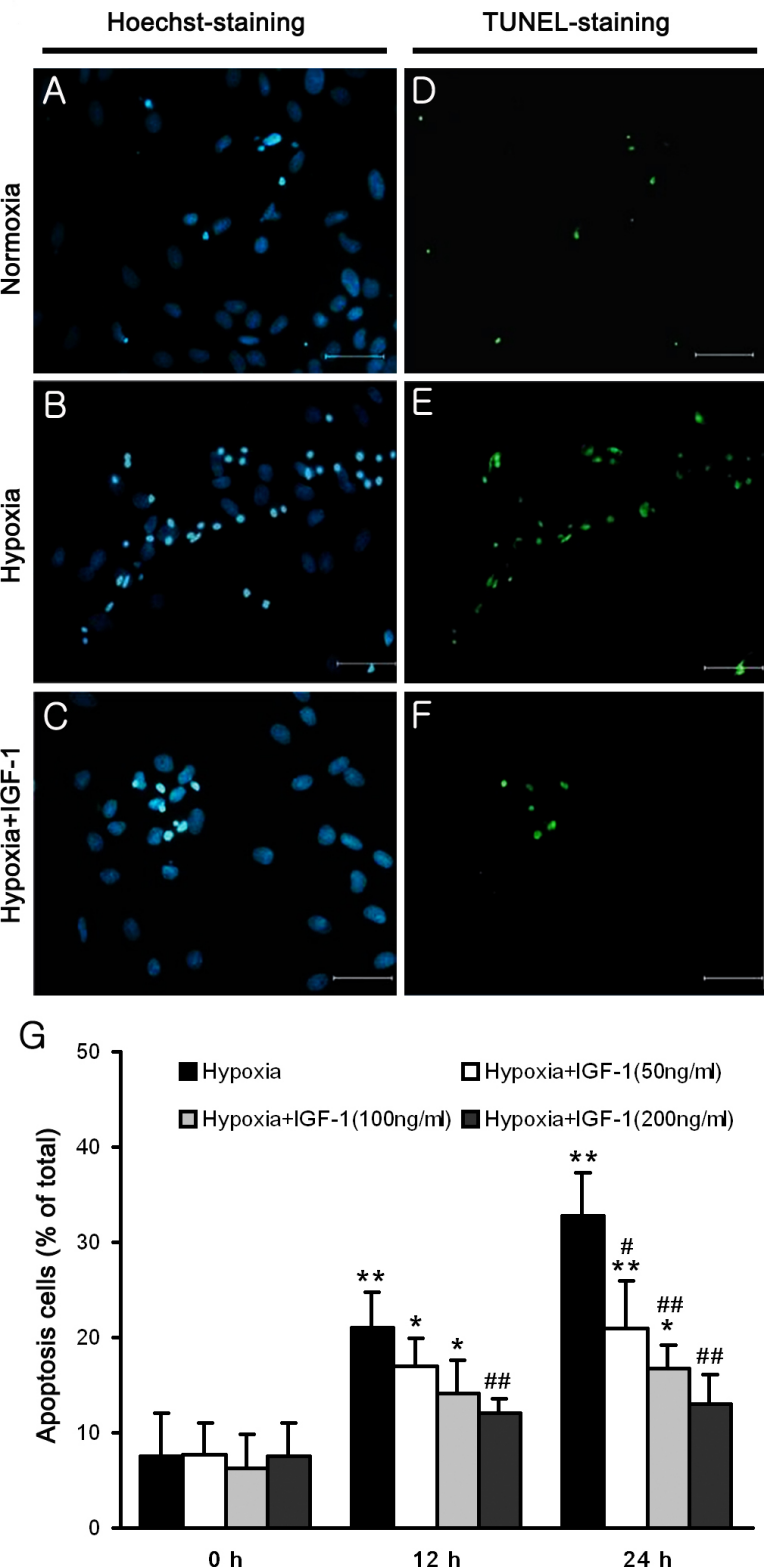
IGF-1 protects primary cultured retinal ganglion cells from hypoxia-induced apoptosis. **A**–**C**: Nuclear morphology is indicated by Hoechst 33342 staining, and (**D**–**F**) DNA breaks were detected by TUNEL analyses. **A**, **D**: Primary cultured retinal ganglion cells (RGCs) were grown with normoxia for control, (**B**, **E**) cells were subjected to hypoxia for 24 h, and (**C**, **F**) cells were incubated with 200 ng/ml IGF-1 and subjected to hypoxia. The scale bar represents 50 μm. **G**: Percentage of apoptotic cells at different time points. Primary cultured RGCs were grown with normoxia or hypoxia. The hypoxia duration 0 h group is the normoxia group. Serial concentrations of IGF-1 (50, 100, and 200 ng/ml) were administered to cultured RGCs before hypoxia was induced to verify the protective effect of IGF-1. TUNEL-positive nuclei were counted in the five non-overlapping fields per coverslip, and then converted to percentages by comparison to the total number of nuclei. The results are expressed as the means ± SEM (standard error of the mean) from three independent experiments. * p<0.05, ** p<0.01 versus the normoxia group; ^#^ p<0.05, ^##^ p<0.01 versus the hypoxia group.

We initially examined the time course of hypoxia-induced apoptosis in primary cultured RGCs to establish a temporal framework for the metabolic and genetic analyses. Nuclear morphology was investigated with Hoechst 33342 staining, and DNA breaks were detected with TUNEL analyses (Figure 2A). In agreement with previous findings [20,31], the hypoxia-induced apoptosis of RGCs was time dependent. In this set of time-course experiments, apoptosis, which was detected as an increase in the number of TUNEL-positive cells observed with fluorescence microscopy, became evident after 12 and 24 h of exposure to a hypoxic environment (p<0.01 versus normoxia group; n=5).

To verify the protective effects of IGF-1 on hypoxia-exposed RGCs, serial concentrations of IGF-1 (50, 100, and 200 ng/ml) were administered to cultured RGCs before hypoxia was induced. RGCs pretreated with 200 ng/ml of IGF-1 exhibited a significant decrease in apoptosis after 12 and 24 h of exposure to a hypoxic environment (p<0.01 versus hypoxia group; n=5). Although no significant improvement was detected with 50 or 100 ng/ml of IGF-1 administration after 12 h of exposure to hypoxic conditions (p>0.05 versus hypoxia group; n=5), these doses of IGF-1 showed a trend toward a decrease in the number of apoptotic cells after 12 h of hypoxic damage, as shown in Figure 2B. These results suggest that IGF-1 suppresses hypoxia-induced damage to RGCs in a dose-dependent manner. Since there were no significant differences in cell apoptosis when the RGCs pretreated with 200 ng/ml under normoxia and hypoxia conditions, this concentration was used in subsequent experiments.

**Figure 3:**
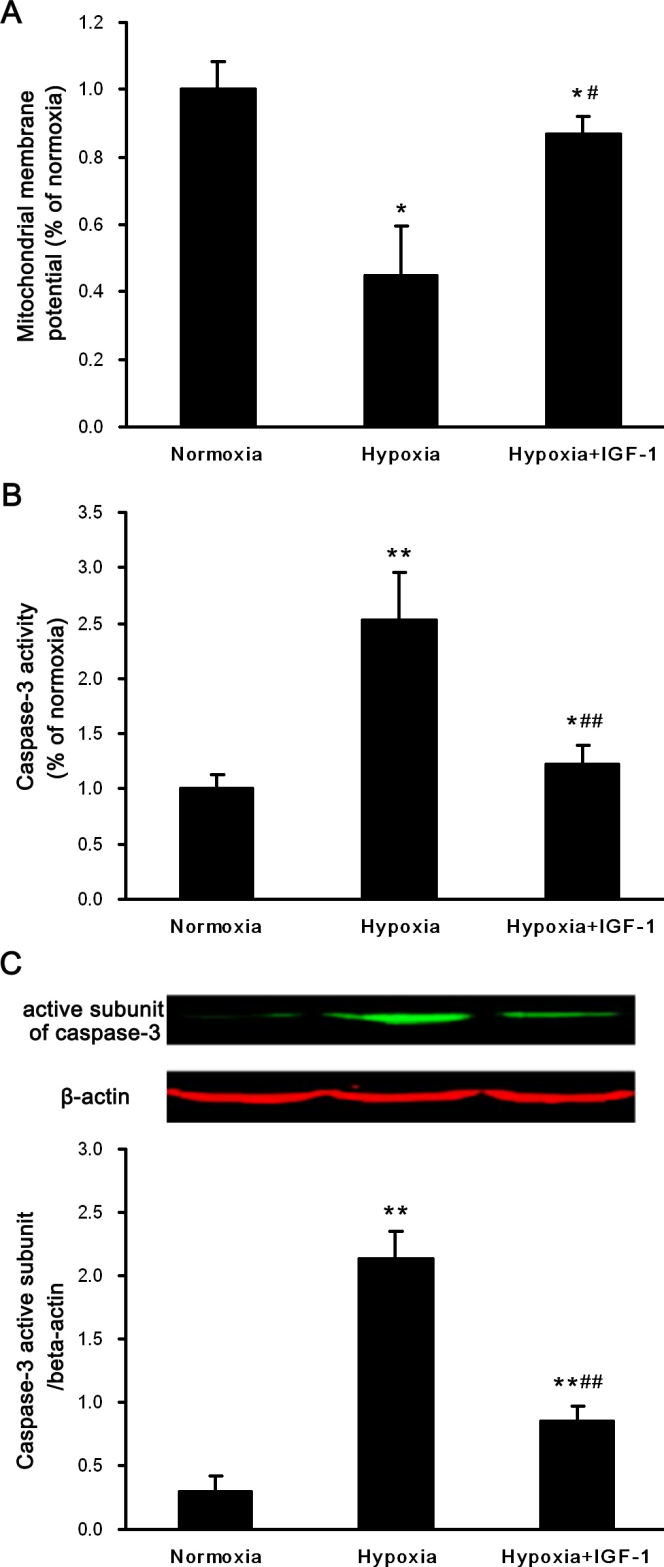
Effects of IGF-1 on the mitochondrial membrane potential and the activity of caspase-3 in primary cultured retinal ganglion cells exposed to hypoxia. Cells were exposed to normoxia or hypoxia for 24 h in the absence or presence of insulin-like growth factor-1 (IGF-1; 200 ng/ml). **A**: JC-1 measured the mitochondrial membrane potential (ΔΨm). **B**: Caspase-3 protease activity in the cells was measured using a caspase-3 colorimetric assay kit. **C**: Western blot analysis for the active subunit of caspase-3 (17 kDa) and β-actin (42 kDa). β-actin immunoreactivity from the same gel is shown as the internal control. The histogram shows the relative protein level normalized to β-actin. Representative results of three independent experiments are shown. The results are expressed as the means ±SEM. * p<0.05, ** p<0.01 versus the normoxia group; ^#^ p<0.05, ^##^ p<0.01 versus the hypoxia group.

The antiapoptotic effects of IGF-1 were further studied using the mitochondrial membrane potential (ΔΨm) and the caspase-3 activation assay. After 24 h of hypoxia exposure, the ΔΨm of the IGF-1-treated and untreated cells was reduced (Figure 3A), although the untreated cells exhibited lower membrane potential values (p<0.05). Western blot analysis showed a substantial increase, approximately sevenfold, in the level of the 17-kDa active subunit of caspase-3 after 24 h of hypoxia (p<0.01 versus normoxia group), and IGF-1 recovered this increase (Figure 3C; p<0.01 versus hypoxia group; n=5). In agreement with the observed protein changes, the activity of caspase-3 was increased 2.5-fold in response to hypoxia in the primary cultured RGCs (p<0.01 versus the normoxia group). This effect was abrogated in the presence of IGF-1 (Figure 3B; p<0.01 versus the hypoxia group; n=5).

### IGF-1 stimulated the Erk-1/2 and Akt pathways in primary cultured retinal ganglion cells

IGF-1 treatment has been shown to activate multiple signaling cascades, including the Erk-1/2 and PI3K/Akt pathways [32]. Erk-1/2 and Akt are well-characterized cell survival signaling pathways, although their responses to hypoxia are cell type-specific. Activated Erk-1/2 and Akt have been shown to mediate antiapoptotic effects through the phosphorylation of Bad and caspase-9, molecular signals that result in the inhibition of caspase-3. Bad is known to be a target of phosphorylation at serine 112 or 136 after Erk-1/2 or Akt is activated, respectively, and this phosphorylation event activates its antiapoptotic properties [33,34]. To investigate whether the Akt and Erk-1/2 signaling pathways and phosphorylated Bad are activated by hypoxia in the presence or absence of IGF-1 treatment in primary cultured RGCs, cells were maintained in a hypoxic environment for 24 h in the presence or absence of IGF-1, and subsequent immunoblot analyses were performed.

**Figure 4:**
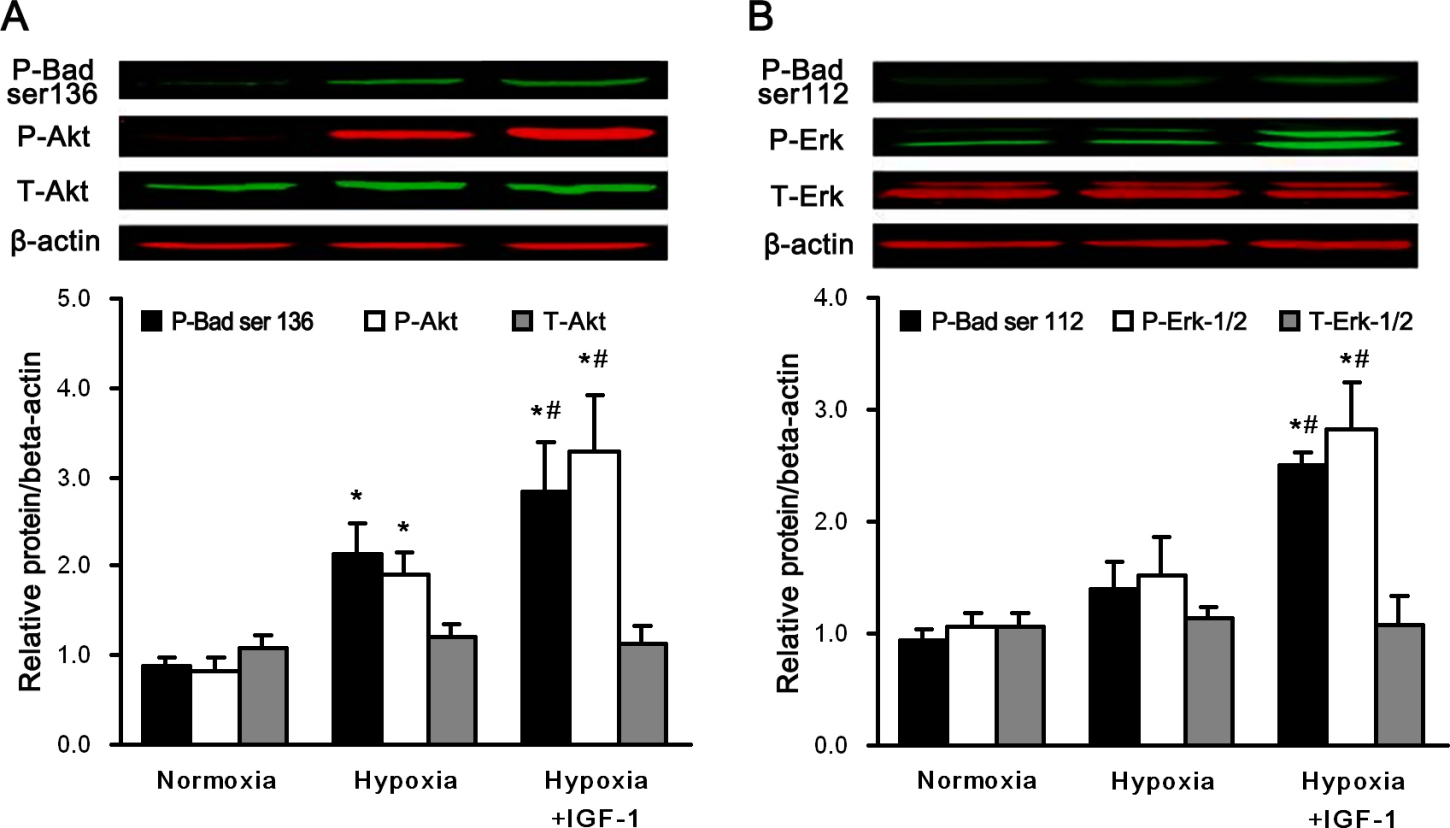
IGF-1 activated the Erk1/2 and Akt pathways in primary cultured retinal ganglion cells. Cells exposed to normoxia or hypoxia with or without IGF-1, 200 ng/ml, for 24 h. Western blot analysis was performed to detect (**A**) the activation of total Akt, phospho-Akt, and phosphorylation of Bad at serine 136 and (**B**) the activation of total Erk-1/2, phospho-Erk-1/2, and phosphorylation of Bad at serine 112. β-actin immunoreactivity from the same gel is shown as the internal control. Histograms show the results of the relative protein level normalized to β-actin. The results are expressed as the means±SEM from three independent experiments. * p<0.05 versus the normoxia group; ^#^p<0.05 versus the hypoxia group.

In the set of experiments shown in Figure 4A, the phospho-Akt and phospho-Bad (Ser136) levels were upregulated after 24 h of hypoxia (p<0.05 versus the normoxia group), and the elevated levels were more significant when the cells were cotreated with IGF-1 (p<0.05 versus the normoxia group, p<0.05 versus the hypoxia group; n=5). Conversely, the total Akt protein levels did not change during that period. Thus, hypoxia and IGF-1 significantly activated the Akt signaling pathway.

Then the effects of IGF-1 on the Erk-1/2 signaling pathway in hypoxic RGC cells were analyzed (Figure 4B). No significant changes were detected in the protein levels of total Erk-1/2, phospho-Erk-1/2, or phospho-Bad (Ser112) after 24 h of hypoxia treatment, which indicated that hypoxia did not activate the Erk-1/2 signaling pathway. However, in the same hypoxic condition, the IGF-1 treatment significantly elevated the levels of phospho-Erk1/2 and phospho-Bad (Ser112; p<0.05 versus the hypoxia group; n=5), indicating that IGF-1 treatment effectively activated the Erk-1/2 signaling pathway.

### The specific inhibitors (AG1024, U0126, and LY294002) reversed the neuroprotective properties of IGF-1

To evaluate whether the activation of the Erk-1/2 and/or the PI3K/Akt signaling pathway is necessary for the IGF-1-induced suppression of apoptosis, we investigated the effect of individually blocking these pathways on the neuroprotective activity of IGF-1 in primary cultured RGCs exposed to hypoxia. We used AG1024, U0126, and LY294002 as specific inhibitors of the IGF-1 receptor, PI3K/Akt, and Erk-1/2, respectively.

**Figure 5:**
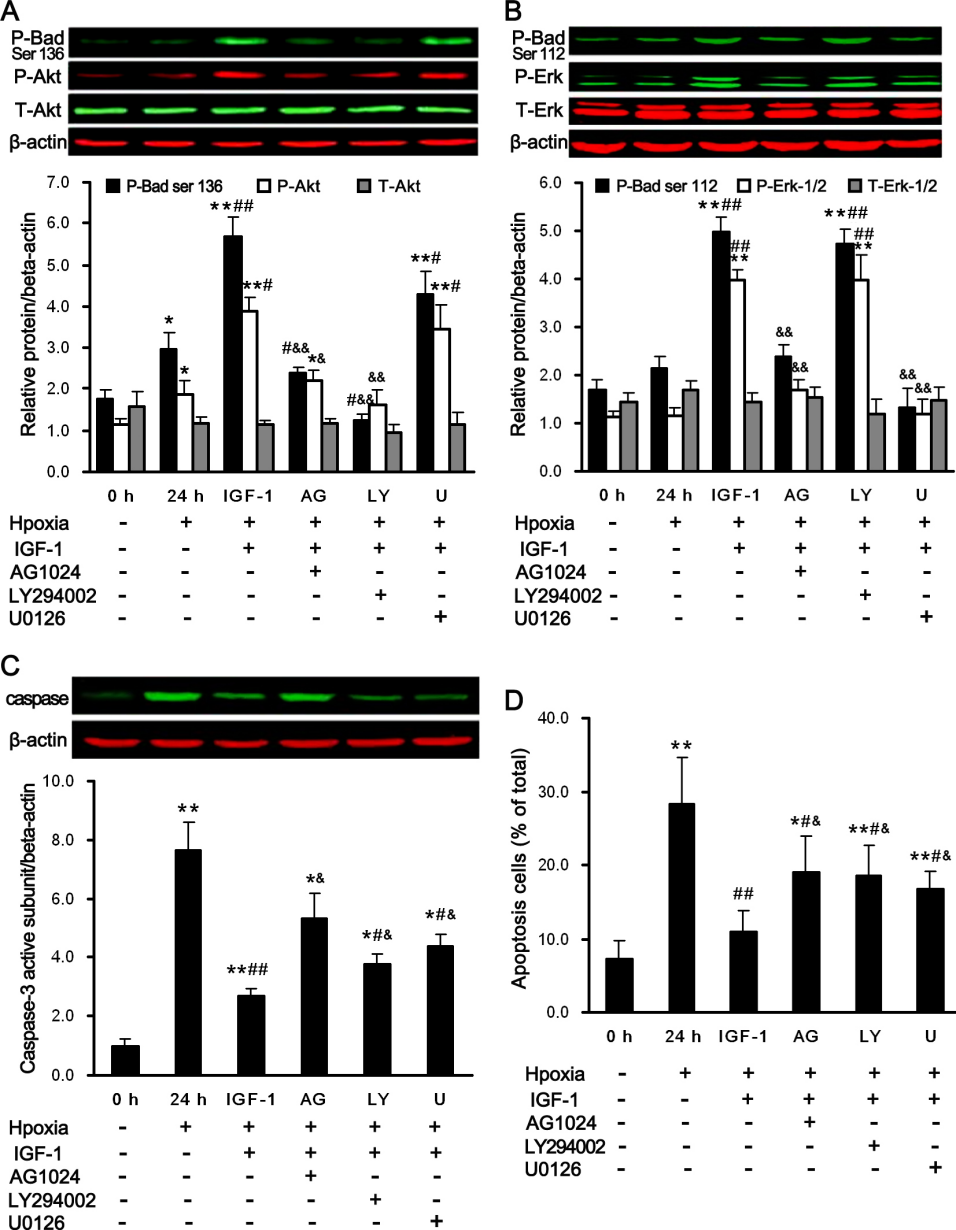
The anti-apoptotic effect of IGF-1 is abrogated by the inhibitors of the IGF-1 receptor (AG1024), Akt (LY294002), and Erk-1/2 (U0126). Retinal ganglion cells (RGCs) were cultured in the absence or presence of U0126 (U, 5 µM), LY294002 (LY, 20 µM), and AG1024 (AG, 10 µM) with or without IGF-1, 200 ng/ml, under normoxia or hypoxia for 24 h. Western blot analysis was performed to detect (**A**) activation of total Akt, and phospho-Akt and phosphorylation of Bad at serine 136, (**B**) activation of total Erk-1/2 and phospho-Erk-1/2, and phosphorylation of Bad at serine 112, and (**C**) the levels of the active capase-3 subunit. β-actin immunoreactivity from the same gel is shown as the internal control. Histograms show the results of the relative protein level normalized to β-actin. **D**: Apoptotic cells were detected TUNEL analyses. Representative results of three independent experiments are shown. The results are expressed as the means±SEM. *p<0.05, **p<0.01 versus the hypoxia group; ^#^p<0.05, ^##^p<0.01 versus the hypoxia group; ^&^p<0.05, ^&&^p<0.01 versus IGF-1 treated group.

The antiapoptotic effect of IGF-1 is believed to be mediated via the IGF-1 receptor. As shown in Figure 5A,B, when hypoxia cells were treated with AG1024 to block the IGF-1 receptors, Erk-1/2 (p<0.05 versus the IGF-1-treated group) and Akt (p<0.05 versus the control group, p<0.05 versus the IGF-1-treated group) were inhibited. This resulted in decreased levels of phospho-Bad (Ser112; p<0.01 versus the IGF-1-treated group) and phospho-Bad (Ser136; p<0.05 versus the hypoxia group, p<0.01 versus the IGF-1-treated group). Moreover, the activity of caspase-3 and TUNEL-positive cells was elevated (p<0.05 versus the control group, p<0.05 versus the IGF-1-treated group; shown in Figure 5C,D).

We then examined the effects of U0126 and LY294002 on the phosphorylation of Erk-1/2 and Akt in the IGF-1-treated RGCs exposed to hypoxic conditions. The hypoxia- or IGF-1-induced upregulation of phospho-Akt was completely suppressed by LY294002 treatment (p<0.01 versus the IGF-1-treated group, shown in Figure 5A). When the PI3K/Akt pathway was inhibited by LY294002, Bad was dephosphorylated at Ser-136 (p<0.01 versus the IGF-1-treated group, shown in Figure 5A), and the levels of TUNEL-positive RGCs and caspase-3 activity were elevated (p<0.05 versus the IGF-1-treated group, shown in Figure 5C,D), although these levels did not reach those observed in hypoxic RGCs. Moreover, no changes in total Akt, Erk-1/2, or phospho-Bad (Ser112) were observed. Similar observations were made for the U0126 treatment, including elevated levels of apoptosis and caspase-3 activity (p<0.05 versus the IGF-1-treated group, shown in Figure 5C,D), and no effects on Akt or phospho-Bad (Ser136) were observed. Conversely, the phosphorylation of Erk-1/2 was completely inhibited, which resulted in the suppression of phospho-Bad (Ser112; p<0.01 versus the IGF-1-treated group, shown in Figure 5B). These results imply that IGF-1 reduces the amount of apoptosis induced by hypoxia in primary cultured RGCs through the activation of the PI3K/Akt and Erk-1/2 pathways.

## Discussion

In this study, we confirmed that hypoxia can induce apoptosis in primary cultured RGCs. We found that IGF-1 protects RGCs against apoptosis induced by hypoxia. The protective effects of IGF-1 for RGCs were significantly reduced by pretreatment with U0126 and LY294002, which are inhibitors of Erk-1/2 and Akt, respectively. These data suggest that the Erk-1/2 and Akt signaling pathways may play a pivotal role in the neuroprotective effects of IGF-1 against hypoxia-induced apoptosis in RGCs.

Clinically, retinal hypoxia underlies several sight-threatening disorders and potentially results in blindness. RGCs show the highest sensitivity to hypoxic challenges [35]. When RGCs are exposed to hypoxic conditions, the lack of oxygen can induce apoptosis in RGCs, thus impairing function [36]. In this study, we revealed that the apoptotic rate in primary cultured RGCs under hypoxia was significantly higher than that in RGCs under normoxia. This observation provides evidence that apoptosis may be the main reason for the loss of RGCs in hypoxia-induced retina injury. IGF-1 is a potent neuronal survival factor and is likely to protect neurons from apoptosis induced by various stimuli [37]. In addition, recent studies have shown that the downregulation of IGF-1 induces apoptosis in RGCs in rats during the early stage of optic nerve injury and that supplementation of IGF-1 can prevent RGC apoptosis and axon elimination in axotomized rats [12,38]. All of these results show that IGF-1 is a key molecule in regulating apoptosis, survival, and neuron regeneration in RGCs. Here, we found that treatment with IGF-1 promoted the survival of RGCs exposed to hypoxia in a dose-dependent manner by attenuating hypoxia-induced apoptosis. Conversely, the neuroprotective effect of IGF-1 can be almost completely eliminated by the IGF-1 receptor antagonist, AG1024. Our findings suggest that IGF-1 exhibits an antiapoptotic action in cultured RGCs subjected to hypoxia.

Apoptosis is regulated by a series of signal transduction cascades consisting of molecules such as the caspase family of proteins, Bcl-2-related proteins, p53, and survivin. Caspases play vital roles in inducing, transducing, and amplifying intracellular apoptotic signals [39]. Caspase-3, one of the most important caspases, is considered the effector and executioner of apoptosis [40]. Our data demonstrate that hypoxia upregulated the activity of caspase-3 in primary cultured RGCs (Figure 3B), suggesting that caspases are involved in hypoxia-induced apoptosis in RGCs. Consequently, we measured not only the enzyme activity but also the protein level of caspase-3 in RGCs after treatment with IGF-1. In agreement with other studies [41-43], our results demonstrated that the increased activation of caspase-3 after hypoxia in cultured RGCs could be effectively inhibited by treatment with IGF-1. Moreover, the effect of IGF-1 on the level of activated caspase-3 could be reversed by AG1024. Therefore, it is likely that IGF-1 protects RGCs from apoptosis, at least in part, by preventing caspase-3 activation.

Previous studies have shown that the Erk-1/2 and/or Akt pathways contribute to the neuroprotective effects of many growth factors by inhibiting caspase-3 [24,44-46]. In this study, we investigated the possible signaling pathways that mediate the protective effects induced by IGF-1 in hypoxic primary cultured RGCs. Western blot analysis showed that IGF-1 induced the phosphorylation of Akt and Erk-1/2 in hypoxic RGCs; these effects were not observed in the presence of AG1024. We found that adding U0126 prevented the phosphorylation of Erk-1/2 induced by IGF-1 and partially inhibited the IGF-mediated antiapoptotic effects. Similarly, the PI3K/Akt inhibitor, LY294002, blocked the IGF-1-induced phosphorylation of Akt, suggesting that PI3K and/or Akt is partially responsible for the neuroprotection induced by IGF-1. Moreover, treatment with either LY294002 or U0126 increased, albeit incompletely, the levels of the active subunit of caspase-3 and RGC apoptosis. Therefore, we conclude that IGF-mediated neuroprotection is associated with the Erk-1/2 and Akt signaling pathways.

It has been reported that there is cross-talk between the MEK/ERK and Akt pathways in non-RGC cell types [47,48]. However, in this study, ERK activation was not affected by the inhibition of Akt, and Akt activation was not altered by inhibition of Erk-1/2 (Figure 5A,B). These findings indicate that ERK and Akt are activated independently and that there is no direct interaction between these two survival signals. Additionally, we found that Akt, but not Erk-1/2, is activated in hypoxic primary cultured RGCs. Our observation is supported by the fact that hypoxia induced a transient activation of Akt in renal epithelial cells and HT1080 cells (human fibrosarcoma) [49,50]. We presume that the activation of Akt in this experimental paradigm would also be a manifestation of a self-defense mechanism against hypoxic insult in RGCs.

There is evidence that growth factors and cytokines cause Erk-1/2 and Akt-dependent cell survival via the phosphorylation of Bad and inhibition of caspase-3 [51,52]. The phosphorylation of Bad may represent an important event in growth factor–induced cell survival [53]. It has been reported that Bad phosphorylation at Ser-136 is promoted by the PI3K/Akt pathway [54], and Bad phosphorylation at Ser-112 has been shown to be controlled by the Erk-1/2 pathway [55]. In agreement with this finding, we have shown that Bad phosphorylation on Ser136 and 112 was elevated when IGF-1 was administered. The elevation can be restored by AG1024, suggesting that IGF-1 significantly phosphorylates Bad at Ser136 and 112. We further investigated whether the phosphorylation of Bad on Ser112 and 136 depended on the PI3K/Akt and Erk-1/2 signaling pathways. U0126 and LY294002, which are specific inhibitors of the Erk-1/2 and PI3K/Akt pathways, respectively, blocked the elevation of phospho-Bad (Ser112) and 136 in RGCs under hypoxia, indicating that the two pathways site-specifically phosphorylate Bad in cultured RGCs during hypoxia.

In conclusion, our ﬁndings demonstrate that IGF-1 exerts a neuroprotective effect against hypoxia-induced RGCs apoptosis. Erk-1/2 and Akt signaling pathways are involved in the potent antiapoptotic effect of IGF-1 in hypoxic conditions. Although further studies are required to determine the therapeutic effect of IGF-1 in protecting RGC viability and neurite outgrowth in vivo, our results present IGF-1 as a novel therapeutic strategy for reducing the vision loss and blindness that result from hypoxia-induced retinal cell loss.
